# The Book World of Medicine and Science

**Published:** 1906-08-04

**Authors:** 


### The Book World of Medicine and Science.

The Test-diet in Intestinal Diseases. By Professor Dr. Adolf Schmidt. Translated from the latest German edition by Charles D. Aaron, M.D. (Philadelphia: F. A. Davis Company. 1906. Pp. 91. Price $1.00 net).

In this essay an account is given of Professor Schmidt's method of examining the fæces as passed under the influence of a standard test-diet and with a view to determine the presence and nature of functional disturbance in the intestine, liver, and pancreas. The motive of the work is to bring such an examination within the range of the practical physician and to place it as readily at his disposal as is already the case in reference to the investigation of the urine and of the contents of the stomach. Professor Schmidt contends that if the scheme which he describes is followed a confident result can be obtained without the use of any complicated apparatus and with the modest expenditure of some ten to fifteen minutes. He claims, further, that such observations carry important diagnostic and therapeutic suggestions and that they are, indeed, essential to the accurate treatment of intestinal disorders. There can be little doubt of the truth of his statement that too little attention is paid to the examination of the fæces, and his work may be expected to stimulate attention to an important, but neglected, collection of facts having considerable clinical significance.

The Nature and Treatment of Cancer: Some Methods of Hypodermic Medication in the Treatment of Inoperable Cancer. By John A. Shaw-Mackenzie, M.D.Lond. Third edition, revised and enlarged. (London: Baillière, Tindall, and Cox. 1906. Pp. 100.)

Dr. Shaw-Mackenzie's little book is now in its third edition, and deserves to be more widely known in the profession. In cases of advanced and inoperable cancer the medical attendant is frequently implored to try something in the way of treatment by the patient and his friends; in this book several methods of treatment are brought forward and discussed, methods which, when carefully and systematically employed, though they can hardly be expected to be curative, may and do relieve many of the symptoms, such as pain, discharge, etc., and prolong the patient's life considerably. We have seen pronounced improvement under trypsin injections, and though the effect produced is partly mental, this is no argument against the employment of such palliative measures, since we have surely accomplished a great deal if by these means we can alleviate the patients' suffering and brighten them up with a ray of hope. Medical men, realising the hopelessness of these cases, are too apt to abandon any systematic line of treatment. It is only by the repeated trial of empyrical remedies such as those mentioned by the author that we may expect at last to discover a curative agent for the disease.

### BOOKS RECEIVED.

Baillière, Tindall, and Cox.

